# Understanding the Relationship between Cotton Fiber Properties and Non-Cellulosic Cell Wall Polysaccharides

**DOI:** 10.1371/journal.pone.0112168

**Published:** 2014-11-10

**Authors:** Dhivyaa Rajasundaram, Jean-Luc Runavot, Xiaoyuan Guo, William G. T. Willats, Frank Meulewaeter, Joachim Selbig

**Affiliations:** 1 Institute of Biochemistry and Biology, University of Potsdam, Potsdam-Golm, 14476, Germany; 2 Max-Planck Institute of Molecular Plant Physiology, Potsdam-Golm, 14476, Germany; 3 Bayer CropScience NV-Innovation Center, Technologiepark 38, 9052 Gent, Belgium; 4 Department of Plant and Environmental Sciences, Faculty of Sciences, University of Copenhagen, Thorvaldsensvej, 40 1.1871, Fredriksberg C, Denmark; USDA-ARS-SRRC, United States of America

## Abstract

A detailed knowledge of cell wall heterogeneity and complexity is crucial for understanding plant growth and development. One key challenge is to establish links between polysaccharide-rich cell walls and their phenotypic characteristics. It is of particular interest for some plant material, like cotton fibers, which are of both biological and industrial importance. To this end, we attempted to study cotton fiber characteristics together with glycan arrays using regression based approaches. Taking advantage of the comprehensive microarray polymer profiling technique (CoMPP), 32 cotton lines from different cotton species were studied. The glycan array was generated by sequential extraction of cell wall polysaccharides from mature cotton fibers and screening samples against eleven extensively characterized cell wall probes. Also, phenotypic characteristics of cotton fibers such as length, strength, elongation and micronaire were measured. The relationship between the two datasets was established in an integrative manner using linear regression methods. In the conducted analysis, we demonstrated the usefulness of regression based approaches in establishing a relationship between glycan measurements and phenotypic traits. In addition, the analysis also identified specific polysaccharides which may play a major role during fiber development for the final fiber characteristics. Three different regression methods identified a negative correlation between micronaire and the xyloglucan and homogalacturonan probes. Moreover, homogalacturonan and callose were shown to be significant predictors for fiber length. The role of these polysaccharides was already pointed out in previous cell wall elongation studies. Additional relationships were predicted for fiber strength and elongation which will need further experimental validation.

## Introduction

Cell walls, the key determinant of overall plant growth and development are primarily composed of polysaccharides, namely cellulose, hemicellulose, and pectins, lignin, and structural proteins [1], [2]. Cell wall biology has been an area of prominent research over many years with the use of novel technologies to probe these higher order structures in the native state. Since the early 1970’s, comparative biochemical analyses revealed that all plant cell walls share several common features. However, they exhibit diversity with respect to their chemical composition [3]–[5]. Indeed, cell walls are structurally complex as they are constantly remodeled and re-constructed during plant growth and development. Also, walls are modulated according to functional requirements, thereby limiting our knowledge on cell wall design [6]–[8]. Biochemical analyses complemented by genetic analyses have identified genes and gene products associated with cell wall synthesis. However, an understanding of how these genes are expressed across cells of different tissues and their impact on cell wall design and maintenance is still lacking [9]–[11]. Furthermore, the glycan-rich cell walls influence the nutritional and processing properties of plant based products such as pulp for paper manufacture, textile fibers, timber products, pharmaceuticals, and materials for fuel and composite manufacture [12]–[15]. Therefore, understanding the plant cell walls is not only fundamental to plant sciences but also of industrial relevance.

Microarrays are widely used in plant research for the high throughput analysis of nucleotides, proteins and increasingly, carbohydrate [16]–[18]. Carbohydrate microarrays also referred to as glycan arrays enable hundreds of glycans to be analyzed in parallel. Glycans on the arrays can include oligosaccharides, polysaccharides, glycoproteins and glycolipids [19]–[21]. Glycan arrays have several biological and medical applications which include glycoproteomic methods to identify new glycoproteins and glycans [22], [23], characterization of glycan probes [24], profiling carbohydrate-lectin interactions [25], [26], glycosaminoglycans-growth factor and cytokine interactions [27], [28], pathogen- induced antibody interaction [29], [30], cancer-antibody induced interaction [31], [32], carbohydrate-virus interactions [33], quantitative carbohydrate-protein interactions [34], and drug discovery [35], [36].

Comprehensive microarray polymer profiling (CoMPP), a microarray based glycan screening method is mostly used for high throughput characterization of plant cell walls. In this technique, generation of microarrays by sequential extraction of cell wall polysaccharides and screening samples against a large number of well-defined cell wall probes such as antibodies, carbohydrate binding proteins and modules is done. This methodology was first described in *Arabidopsis thaliana* and *Physcomitrella patens* [37]. In the study of Singh et al, application of CoMPP to study cotton fibers showed that towards the end of elongation, there was a loss in certain cell wall polymer epitopes [38]. Despite the availability of glycan arrays from several experiments, computational analysis has mostly been restricted to collection of glycobiology information in databases, motif analysis of glycans, and oligosaccharide structure determination [39]–[41].

In our study, we used the glycan array technology to study cotton fibers, one of the most important raw materials for the textile industry. There are four different domesticated species producing cotton fibers namely *Gossypium hirsutum* (‘Upland cotton’), *Gossypium barbadense* (‘Pima‘or ‘Egyptian‘cotton), *Gossypium arboreum* (‘Tree cotton‘), and *Gossypium herbaceum* [42]. The development of cotton fibers occurs in four major stages: initiation, elongation, secondary wall synthesis and maturation. Although much work has already been done on the cotton fiber transcriptome, the key question in cotton fiber research is to link the cell wall profile of different cotton types to the cotton fiber properties and to a better understanding of fiber development [43]–[46]. Here, we aim to study the relation between fiber properties and non-cellulosic polysaccharide composition using univariate and multivariate regression based approaches on a diverse set of cotton fibers. To this end, we analyzed two datasets for the same cotton fibers: a glycan array profile and the physical fiber properties as determined by HVI and AFIS. We elucidated the usefulness of regression based approaches to determine the functional relationship between the two datasets and we also selected a subset of variables which have a good prediction of the phenotypic traits.

## Materials and Methods

### Plant material and evaluation of phenotypic traits

In this study, we used 32 different cotton lines of which three are from *Gossypium arboreum*, three from *Gossypium barbadense*, two from *Gossypium herbaceum* and 24 from *Gossypium hirsutum*. The cotton lines used in this study are listed in Table S1, including the plant introduction number (PI number) from the USDA National Plant Germplasm System (http://www.ars-grin.gov/npgs/). Seeds were sown in soil compost and plants were grown at constant conditions in a greenhouse at 26–28°C during a 16 h photoperiod. Mature cotton fibers were collected by harvesting all fully open bolls from several plants. The impact of boll position and plant-to-plant variation was minimized by mixing the fiber from all harvested bolls. Two types of analyses were performed on these fibers, the first being the glycan array measurements (Table S2) and the second being fiber characteristics/phenotype measurements (Table S1). For each line, High Volume Instrument (HVI) and Advanced Fiber Information System (AFIS) measurements were performed on 40 g of mature cotton fiber by CIRAD (France) according to the standard methods ASTM D3818-92 and D5867-05. These measurements were done on 6 and 5 replicates for HVI and AFIS, respectively, except for micronaire where only 2 replicates were performed. Five fiber characteristics which include length from HVI and AFIS, strength, elongation and micronaire were selected for further analysis due to their importance for textile processing. Length HVI refers to the average fiber length of the longer 50% of fibers in a given sample. Length AFIS (W) L deduces length parameters from individual fiber measurements. Strength of the cotton fiber refers to the force required to break a bundle of fibers 1 tex in size (1 tex equals the weight in grams of 1000 meters of fibers). Elongation of the cotton fibers is the measurement of the elasticity of cotton fibers with a higher number indicating more elasticity. Micronaire is obtained by measuring the resistance of the fibers to airflow and depends on the fiber fineness and degree of maturation.

### Comprehensive Microarray Polymer Profiling (CoMPP) of mature cotton fiber cell wall

CoMPP analysis was performed on mature cotton fibers as previously described by [38] with minor modifications. Mature cotton fiber samples were extracted sequentially in 50 mM cyclohexanediamine tetraacetic acid (CDTA) and 4 M Sodium hydroxide (NaOH) with 1% (v/v) sodium tetrahydridoborate (NaBH_4_). These two solvents were used to extract pectins and non-cellulosic polysaccharides, respectively. For each line, 300 ul of solvent was added to 10 mg of sample and incubated with shaking for 2 h. After centrifugation, supernatant from each extraction was printed in four replicates and four dilutions (1∶2, 1∶6, 1∶18 and 1∶54 [v/v] dilutions). Cadoxen extraction was omitted because it is mainly used to extract cellulose which we do not aim to analyse in our study. The array was probed with eleven monoclonal antibodies (mAbs) recognizing different carbohydrate epitopes as listed out in Table 1. A heat map was generated to display the relative intensity of each signal to the maximum signal observed within each antibody detection (Table S2). CoMPP is a semi-quantitative technique and should not be taken to obtain absolute amounts. Practically speaking, we set the maximum value in the whole data sheet as 100 and the other values are divided by this maximum value and multiplied by 100 to obtain numbers comprised between 0 and 100. When the quantification is done, the arrays are manually checked to make sure that there are clear dots on it and not only background or noise. The negative control is an array incubated with 5% milk in PBS and probed with secondary antibody and then developed as the others.

**Table 1:** List of probes used in the glycan array.

Probes used in the analysis	Specificity of the probes	Reference	Source
BS-400-2	(1,3)-β-D-glucan (callose)	[47]	Purchased from Biosupplies (Australia)
JIM5	Partially methyl-esterified homogalacturonan (HG)	[48]	Paul Knox lab
LM19	Un-esterified homogalacturonan (HG)	[49]	Paul Knox lab
JIM13	Arabinogalactan (AGP)	[50]	Paul Knox lab
JIM20	Extensin glycoproteins	[51]	Paul Knox lab
LM11	Xylan	[52]	Paul Knox lab
LM15	XXXG xyloglucans (XG) epitope	[53]	Paul Knox lab
LM24	XXLG and XLLG xyloglucan (XG) epitopes	[24]	Paul Knox lab
LM25	XXLG and XLLG xyloglucan (XG) epitopes	[24]	Paul Knox lab
BS-400-4	Mannan	[54]	Purchased from Biosupplies (Australia)
LM21	Mannan	[55]	Paul Knox lab

### Pre-processing of the data

For the statistical analysis, we use R version 3.1.0 on a 64 bit linux platform [56]. The numerical values from both datasets were of different physical quantities and on different scales of magnitude. Moreover, there is no external knowledge that variables with higher numeric variation should be considered more important. Standardization of the raw data was done by computing z- scores of the raw data. Z- scores were calculated for each data point by subtracting the mean and dividing by the standard deviation of all data points.

### Linear methods to delineate the relationship between the two datasets

Multiple regression models the relationship between a single scalar response variable and a set of explanatory (or independent) variables. Here, we used multiple regression analysis to model which of the cell wall probes were associated to the fiber characteristics. This allowed us to determine the overall fit (variance explained) of the model and the relative contribution of each of the cell wall probes to the total variance explained. The results from the analysis were reported in the coefficients and ANOVA tables. Summary of the fitted model object gave an account of the residuals, the estimates of the intercept, the slope (with the results of a t-test), the residual standard error, the *R^2^* statistic and the results of an F-test. The terms used in the output of regression analysis are defined as follows: residual standard error is the standard deviation of the data about the regression line. The squared multiple correlation coefficient (R^2^) is the proportion of variability in the response that is fitted in the model and the F value is a test statistic to decide whether the model as a whole has statistically significant predictive capability. p values give the statistically significant predictive capability in the presence of other variables [57], [58]. Based on this, five models were selected to determine which of the cell wall polysaccharides play an important role in determining that particular fiber characteristic.

In addition to the multiple regression analysis, relationships between multiple dependent and independent variables were investigated simultaneously using canonical correlation analysis (CCA). The two sets of data were represented by matrices X (dimension *n*×*p)* and Y (dimension *n*×*q)* and columns in X and Y denote the variables p (glycan measurements) and q (fiber characteristics) respectively. Classification of variables as dependent or independent is of little importance for the statistical estimation of the canonical functions as canonical correlation finds linear combinations of sets of multiple dependent and independent variables which are maximally correlated [59], [60].

The first step in CCA was to derive one or more canonical function between the glycan and phenotypic measurements. Each function consisted of a pair of variates, one representing the cell wall probes and the other representing the fiber characteristics. The maximum number of canonical variates (functions) that could be extracted from the sets of variables equals the number of variables in the smallest data set, independent or dependent. As a result, the first pair of canonical variates was derived so as to have the highest intercorrelation possible between the glycan array and the fiber measurements. Technically, the second pair of canonical variates exhibits the maximum relationship between the two sets of variables (variates) not accounted for by the first pair of variates and successive pairs of canonical variates were based on residual variance. Therefore each of the pairs of variates is orthogonal and independent of all other variates derived from the same set of data. The strength of the relationship between the pairs of variates obtained from both datasets was determined by the canonical correlation. An estimate of shared variance between the canonical variates was provided by the squared canonical correlations, also called canonical roots or eigenvalues. The statistical significance of each canonical function was assessed using multivariate tests of significance namely Wilk’s lambda, Hotelling’s trace, Pillai’s trace and Roy’s greatest characteristic criterion (Roy’s gcr). The statistically significant canonical functions were then interpreted using canonical loadings, cross-loadings and redundancy index [61]–[65]. We used the “mixOmics” package [66] in R to perform the canonical correlation analysis.

### Sparse partial least square regression to predict the cell wall probes associated to fiber characteristics

Partial least squares (PLS), a well-known regression technique dealing with collinear matrices, clearly has an edge over other regression techniques [67]. Unlike CCA, the PLS latent variables are linear combinations of the variables based on the maximization of covariance but do not allow feature selection. There are many variants of PLS of which we focused on a sparse partial least squares approach (sPLS) which includes a built-in feature to select variables while integrating the data. We used the “mixOmics” package [66] in the regression mode. Specifically, we use a two block data setup, X be the *n*x*p* matrix and Y be the *n*x*q* matrix where n denotes the samples, variables p and q denote the glycan measurements and fiber characteristics respectively. Sparse PLS, based on lasso regression penalizes the loading vectors using singular value decomposition and has an additional advantage to perform better even when the covariates are highly correlated. We used sPLS in the regression mode and the aim was to model the relationship between the variables and also predict one group of variables from the other [68]–[71].

## Results

### Standardization of the raw data

In this study, we attempted to assess the relationship between the cell wall polysaccharides and the physical fiber properties of mature cotton fibers, the data of which are provided as Table S1 and S2. The glycan array values used for the regression analysis were the sums from the CDTA and the NaOH extractions as performing the analysis using the individual values gave the same correlations. For the fiber characteristics dataset, the values were in different units and scales such as mm (for length), g/tex (for strength), and percentage (for elongation). To make the fiber characteristics dataset compliant to the glycan array, the raw data were jointly standardized using z scores prior to the analysis.

### Modelling the fiber properties using linear regression models

We investigated the linear relationship between the fiber properties and their corresponding array values by a series of regression analyses. Multiple regression models were built considering one fiber characteristic at a time as the dependent variable and multiple probes as the independent variables. Five such models were predicted for the phenotypic traits and the overall model prediction result (Table 2) shows that the model for length HVI, length AFIS and micronaire are statistically significant. The significant predictor variables of length HVI are BS-400-2, LM19 and the ones for length AFIS include BS-400-2, JIM5, JIM20, LM15, and LM19. LM15, LM19, LM24 and LM25 are the significant predictor variables for the model predicting cotton fiber micronaire and the overall model has a p value of 4.906e-06. The models for strength and elongation do not show any statistical significance.

**Table 2:** Summary statistics of the five possible multiple regression models.

Fibercharacteristics	Residual standarderror	Multiple R-squared	Adjusted R-squared	F-statistic	p-value	Significantpredictors
Length HVI	0.696	0.706	0.545	4.372 on 11 and 20 DF	0.002	BS-400-2, LM19
Length AFIS	0.632	0.720	0.566	4.677 on 11 and 20 DF	0.001	BS-400-2, JIM5, JIM20,LM15, LM19
Strength	0.940	0.378	0.036	1.107 on 11 and 20 DF	0.404	JIM20
Elongation	0.825	0.376	0.033	1.098 on 11 and 20 DF	0.410	–
Micronaire	0.469	0.851	0.769	10.4 on 11 and 20 DF	4.906e-06	LM15, LM19, LM24,LM25

### Assessing the relationship between multiple probes and all of the fiber characteristics simultaneously using canonical correlation analysis

The multiple regression analysis can predict the value of a single (metric) dependent variable from a linear function of a set of independent variables. However, to explore the relationship of sets of multiple predictor variables (probe measurements) to sets of multiple response variables (phenotypic traits) CCA was used. As CCA uses information from all the variables in both the predictor and response sets, it serves as a more efficient approach than methods routinely used, such as multiple linear regression.

**Figure 1:**
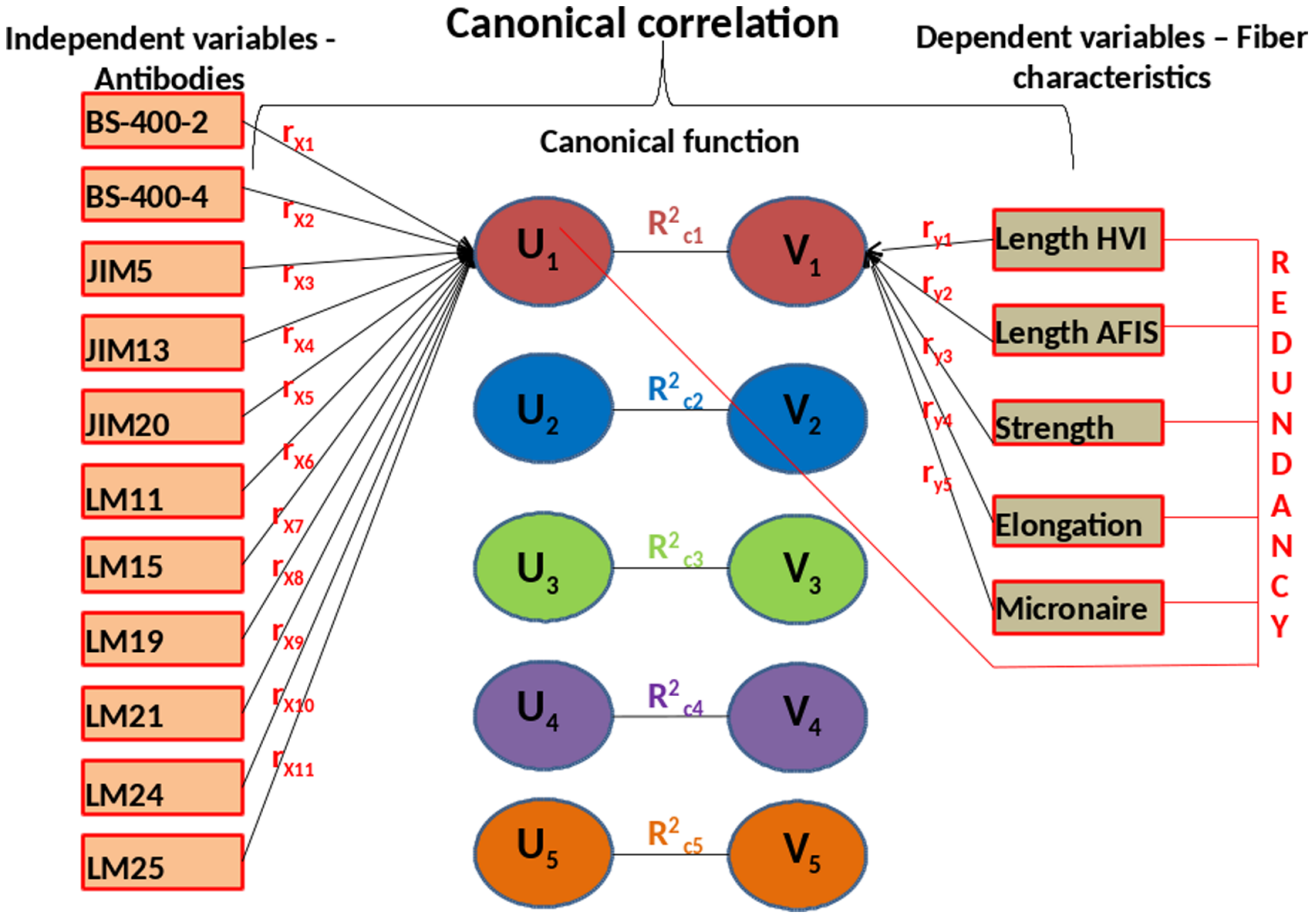
Canonical correlation analysis maximizes the correlation between the linear combination of the cell wall polysaccharides in the glycan array and the fiber properties. In this figure, given a linear combination of *X* variables: *U_1_* = *f*_1×1_+ *f*_2×2_+ …+*f*_p_ *X*_p_ and a linear combination of *Y* variables: *V_1_* = *g*_1_ *Y*_1_+ *g*_2_ *Y*_2_+ …+*g*_q_ *Y*_q_, the first canonical correlation is the maximum correlation coefficient between *U_1_* and *V_1_*, for all *U_1_* and *V_1_.*

For the CCA analysis, the glycan array measurements (probed by 11 antibodies) are designated as the set of independent variables. The fiber characteristics namely length AFIS, length HVI, strength, elongation and micronaire were specified as the set of dependent variables (Figure 1). However, it is of little importance to classify the variables as independent or dependent as the technique aims to maximize the correlation between the two sets of variables. In Figure 1, the terms r_x1_ to r_x11_ represent the canonical loadings which reflect the variance that the eleven variables from the glycan array shares with the independent canonical variate U_1_. Similarly the terms r_y1_ to r_y5_ represent the canonical loadings which reflect the variance that the five phenotypic variables share with the dependent canonical variate V_1_. The canonical correlation between the independent and dependent canonical variates is measured by the canonical functions which are represented by R^2^_c1_ to R^2^_c5_. The statistical problem involved identifying any latent relationships (relationships between composites of variables rather than the individual variables themselves) between the glycan and the fiber measurements.

The canonical correlation which is based on the linear relationship of the glycan array data and fiber characteristics was computed to derive five canonical functions. Each of these functions consists of a pair of variates, one for the glycan array data and the other for the fiber characteristics. Since the study includes 11 independent variables and 5 dependent variables, the maximum number of canonical functions which could be derived is five (Table 3).

**Table 3:** Canonical Correlation analysis relating probe signals and fiber characteristics with the measure of overall model fit.

Canonical function	Canonical correlation	Canonical R^2^	F statistics	p-value
1	0.945	0.883	2.85	1.57338e-05
2	0.868	0.753	1.88	1.072479e-02
3	0.803	0.645	1.30	2.035076e-01
4	0.523	0.273	0.59	8.715784e-01
5	0.342	0.116	0.34	9.040993e-01

In addition to tests of each canonical function separately, multivariate tests of these five functions simultaneously were also performed. The test statistics employed include Wilks’ lambda, Pillai’s criterion, Hotelling’s trace, and Roy’s gcr. Table 4 details the p-values from the multivariate test statistics, which all indicate that only the first canonical function, taken collectively, is statistically significant at 1% level.

**Table 4:** Multivariate tests of significance for the canonical functions.

Canonical function	Wilks' Lambda, using F-approximation (Rao's F):	Hotelling-Lawley Trace, using F-approximation:	Pillai-Bartlett Trace using F-approximation	Roy’s largest root using F-approximation
**1**	1.57338e-05	2.666759e-07	0.00	8.732348e-12
**2**	1.072479e-02	1.221924e-03	0.042	
**3**	2.035076e-01	5.475660e-02	0.285	
**4**	8.715784e-01	8.365373e-01	0.801	
**5**	9.040993e-01	8.855773e-01	0.848	

From the results of these tests, we proceeded to interpret other aspects of the analysis based on the first canonical function. A redundancy index was calculated for the independent and dependent variates of the first function in Table 5. The redundancy index is calculated as the average loading squared times the canonical R^2^. As can be seen, the redundancy index for the dependent (0.191) and independent variates (0.200) is quite low. The low values result from the relatively low shared variance in the dependent variates (0.214) and independent variates (0.225), not the canonical R^2^. With such a small percentage, this is an example of a statistically significant canonical function that does not have practical significance because it does not explain a large proportion of the dependent variables’ variance.

**Table 5:** Redundancy analysis of dependent and independent variates for the first canonical function.

Standardized variance of the dependent variables explained by				
Their own Canonical variates (shared variance)			The opposite canonical variates (Redundancy)	
Percentage	Cumulative percentage	Canonical R^2^	Percentage	Cumulative percentage
0.214	0.214	0.883	0.191	0.191
**Standardized variance of the independent variables explained by**				
**Their own Canonical variates (shared variance)**			**The opposite canonical variates (Redundancy)**	
**Percentage**	**Cumulative percentage**	**Canonical R^2^**	**Percentage**	**Cumulative percentage**
0.225	0.225	0.883	0.200	0.200

The interpretations involve examining the canonical functions to determine the relative importance of each of the original variables in deriving the canonical relationships (Table 6). The three methods for interpretation are (1) canonical weights (standardized coefficients), (2) canonical loadings (structure correlations), and (3) canonical cross-loadings.

**Table 6:** Canonical weights, loadings, and cross-loadings for the first canonical function.

	Canonical weights	Canonical loadings	Canonical cross-loadings
**Dependent variables**			
**Length HVI**	−0.636	0.127	0.120
**Strength**	−0.226	0.040	0.038
**Elongation**	−0.033	0.056	0.053
**Micronaire**	−0.843	−0.941	−0.890
**Length AFIS**	0.810	0.409	0.387
**Independent variables**			
**BS-400-2**	0.184	0.362	0.342
**BS-400-4**	−0.487	−0.119	−0.113
**JIM5**	−0.823	0. 0.632	0.598
**JIM13**	−0.290	0.288	0.272
**JIM20**	−0.204	−0.376	−0.355
**LM11**	−0.114	−0.360	−0.340
**LM15**	−0.719	0.638	0.603
**LM19**	1.243	0.712	0.672
**LM21**	0.356	0,275	0.260
**LM24**	−0.324	0.066	0.062
**LM25**	1.082	0.767	0.724

Table 6 contains the standardized canonical weights for each canonical variate for both dependent and independent variables. As mentioned earlier, the magnitude of the weights represent their relative contribution to the variate. Based on the size of the weights, the order of contribution of independent variables to the first variate is LM19, LM25, JIM5, LM15, BS-400-4, LM21, LM24, JIM13, and JIM20 and the dependent variable order on the first variate is micronaire followed by length AFIS, length HVI, strength and elongation. Because canonical weights are typically unstable, particularly in instances of multicollinearity, owing to their calculation solely to optimize the canonical correlation, the canonical loading and cross-loadings are considered more appropriate.

Table 6 also contains the canonical loadings for the dependent and independent variates for the first canonical functions. In the first dependent variates, all the five variables had different values of loadings resulting in low shared variance (0.214). This indicates a low degree of inter-correlation among the five dependent variables. Observing the independent variates, there is a different pattern and loading values ranged from 0.06 to 0.77. The variables with the highest loadings on the independent variate are LM25, LM19, LM15, and JIM5. We also observed some loadings with negative values which include those of BS-400-4, JIM20, and LM11.

In case of the cross loadings, micronaire has a value of −0.890 and interestingly has a negative loading. Length AFIS to some extent has a loading value of 0.387 while those of the other variables is low. By squaring these terms, we find the percentage of the variance for each of the variables explained by function 1. The results show that 79.21 percent of the variance in micronaire, 14.97 percent of the variance in length AFIS is explained by function 1 whereas strength, elongation and length HVI have very low values. Similarly for the independent variables’ cross loadings, variables LM25, LM19, LM15, JIM5 have high correlations of 0.73, 0.67, 0.61, and 0.61 respectively. From this information, approximately 51.8% of the variance in LM25, 45.1% of the variance in LM19, 36.3% of the variance in LM15, and 35.7% of the variance in JIM5 is explained by the dependent canonical variates.

The final step of interpretation is examining the signs of the cross-loadings. Examining the signs of the independent variables’ cross loadings, those with high correlations have a positive direct relationship whereas BS-400-4, JIM20 and LM11 have an inverse relationship. The four highest cross-loadings of the first independent variate correspond to the variables with the highest canonical loadings as well. Observing the cross loadings of the dependent variables, we see that micronaire has the highest canonical loading and an inverse relationship. Also, elongation is observed to have an inverse relation but since it is of very low value, it was not taken into account.

### sPLS approach to predict specific cell wall polysaccharides involved in fiber properties

**Figure 2:**
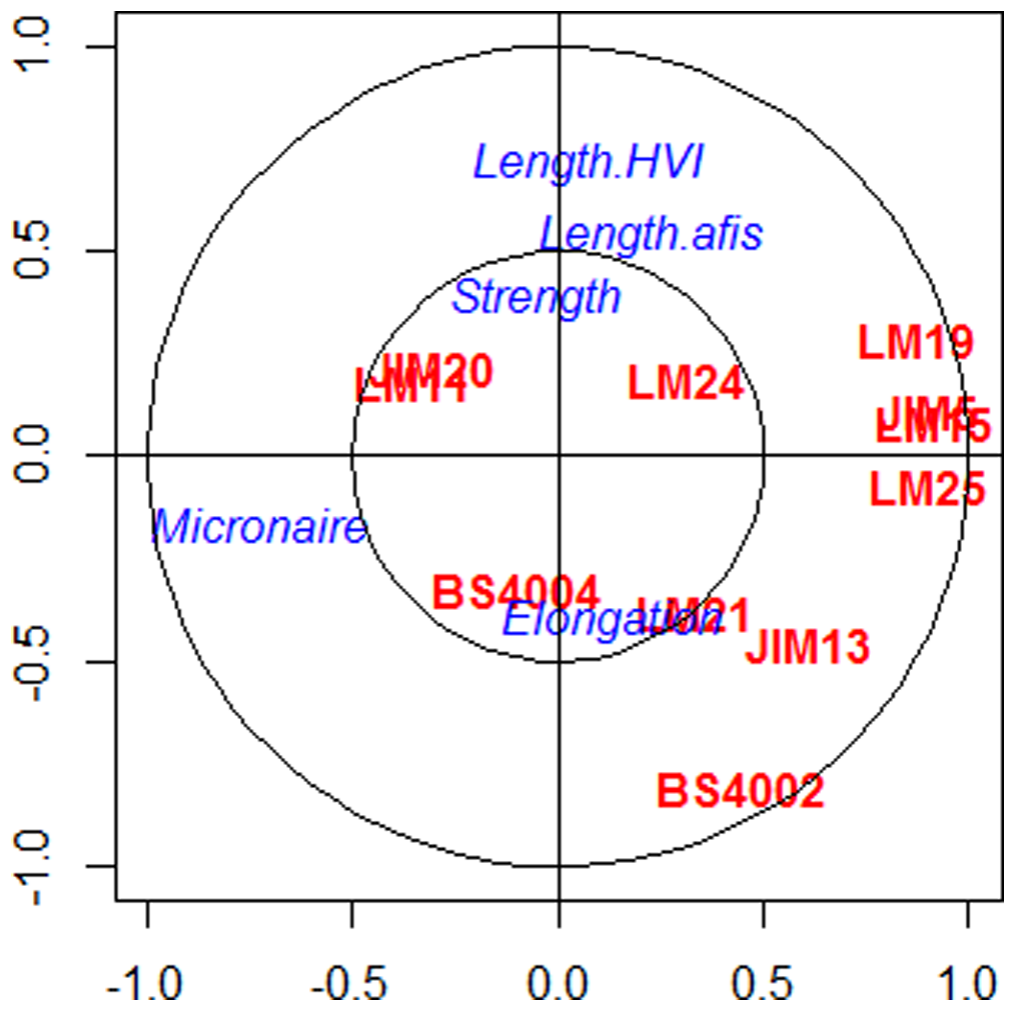
Graphical representation of the variables selected by sPLS on the first two dimensions predicts specific cell wall polysaccharides linked to the fiber properties. The coordinates of each variable are obtained by computing the correlation between the latent variable vectors and the original dataset. The selected variables are then projected onto correlation circles where highly correlated variables cluster together. These graphics help to identify association between the two datasets. The correlation between two variables is positive if the angle is sharp cos(α)>0, negative if the angle is obtuse cos(θ)<0, and null if the vectors are perpendicular cos(β)∼0.

sPLS was computed in the regression mode and the input for the analysis included the 11 cell wall probes along with the five fiber characteristics The number of dimensions *H* to be retained was estimated with the Qh^2^ criterion, for which a value below the threshold 0.0975 indicates a significant contribution for the prediction purpose. The Qh^2^ values calculated for each dimension of the sPLS showed that 2 dimensions were enough to capture the whole information. From Figure 2, we can interpret the results from the sPLS via the correlation circle plot where the predictor variables are in red and the response variables are represented in blue. A correlation circle plot is a graphic tool to represent variables of two different data-types and examine the relationships between the variables and variates. In this plot, variables namely cell wall probes and fiber measurements can be represented as vectors. The relationship between these two data-types is approximated by the inner product between the associated vectors which is defined as the product of the two vector lengths and their cosine angle. For better interpretation, two circles of radii 0.5 and 1 are represented to visualize the variables. The longer the distance to the origin, the stronger is the relationship between the variables.

Using the interpretation which is detailed, we find that BS-400-4, LM21, and JIM13 share a positive relationship with elongation characteristic of cotton fibers. We were also able to attribute the strength of the cotton fibers to JIM20, LM11 and LM24. Interestingly, LM19, JIM5, LM15, and LM25 were projected diametrically opposite to that of the micronaire in the correlation circle, thereby indicating a strongly negative relationship. Length HVI and length AFIS share a negative relation to BS-400-2. To estimate the significance of the predicted relationships, the root mean squared error prediction (RMSEP) values were computed for each response variable (fiber properties) and ranked according to the absolute value of their loadings in v_2_. The lower the RMSEP value, the better the prediction of the model is. In this case, the model for micronaire was the best one (RMSEP of 0.71), followed by that of length AFIS (1.13), strength (1.14), elongation (1.15), and length HVI (1.21).

**Figure 3:**
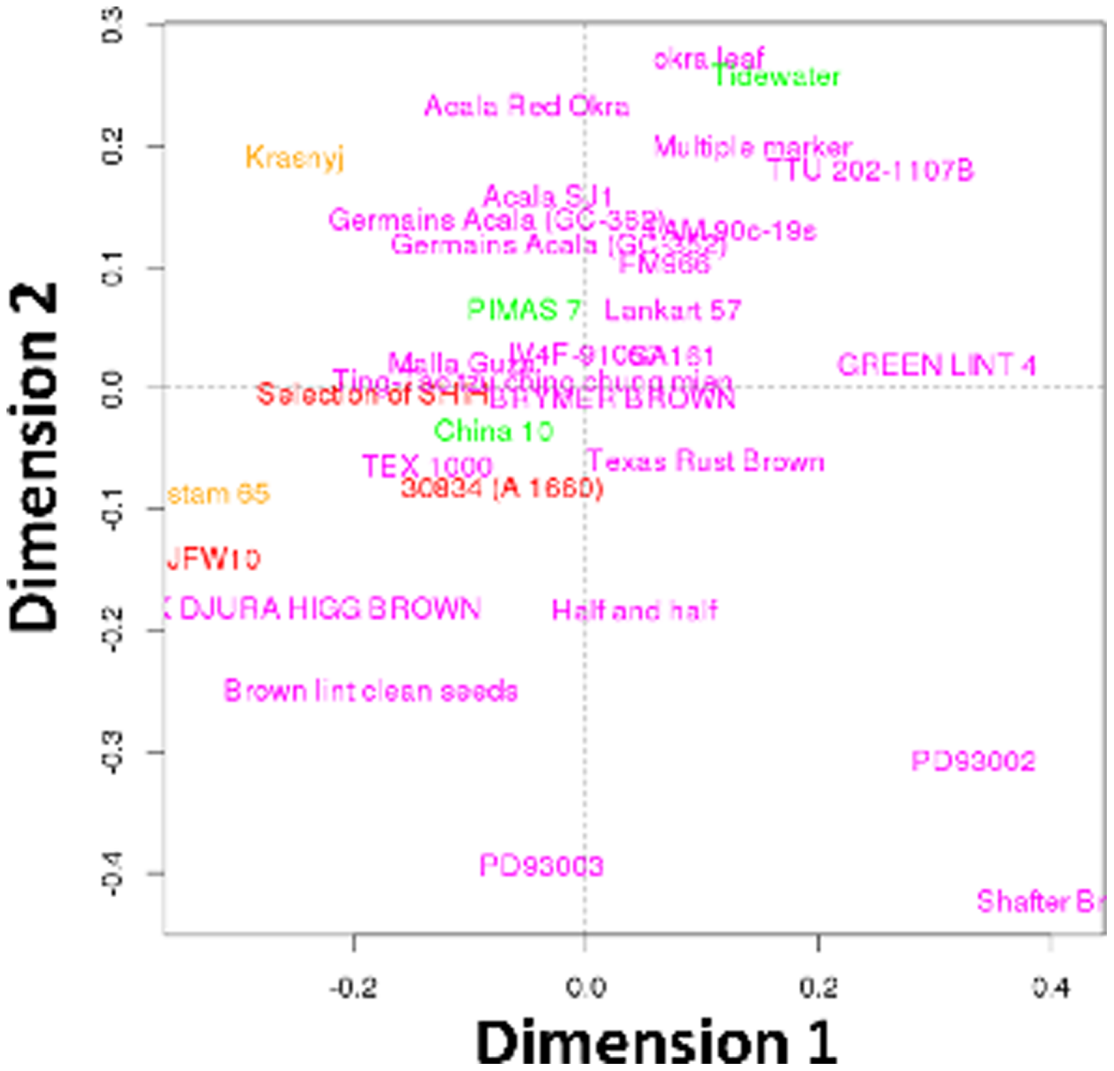
Graphical representation of the cotton lines on the first two sPLS dimensions shows the trend in clustering of specific cotton lines across different species. Four different species of cotton are shown in different colors. *Gossypium hirsutum* is colored in magenta, *Gossypium barbadense* in green, *Gossypium herbaceum* in orange and *Gossypium arboreum* in red.

Figure 3 displays the graphical representation of the cotton lines in dimension 1 and 2. This plot shows that some of the lines are clustered together, with Acala SJ1, Germains Acala (GC 352 and GC 362), TAM-90C-19 S, and FM966 forming one cluster, Acala red okra, okra leaf, multiple marker, Tidewater, and TTU 202-1107B forming a second cluster and PIMAS7, Lankart 57, IV4F-91057, GA161, Ting tao tzu ching chung mien, Brymer brown, Malla guza, Selection of SHIH, China 10, Texas rust brown, Tex 1000 and 30834 (A1660) forming a third cluster. Strikingly, some of these clusters contain lines from different *Gossypium* species and lines from one species often belong to multiple clusters. The variation in fiber characteristics and composition is thus clearly not species-specific. However, one should be careful in interpreting the results from the individual lines as the study was designed to discover correlations between fiber properties and composition and not to study properties of individual lines.

## Discussion

Understanding the genetics and physiology of cotton fibers is of importance to the textile industry. There have been numerous studies, both profiling and sequencing based experiments to study cotton fiber development at the transcriptional level. The high degree of transcriptional complexity in the development of cotton fibers has been the focus of these studies [43], [72]–[75]. We used the CoMPP technique in our analysis to study directly the glycan composition of cotton lines from different species. The work presented here demonstrates the potential of glycan microarrays in combination with multivariate statistical approaches for understanding the cell wall composition responsible for the fiber characteristics. Specifically, the use of regression based approaches in our study helps to predict models for each of the fiber trait under study.

We studied the association between glycan array measurements and their relation with fiber characteristics using linear approaches like multiple regression, CCA and sPLS. From the results of multiple regression (Table 2), we were able to predict three models for length HVI, length AFIS and micronaire of cotton fibers but not for strength and elongation characteristics. Moreover, to extend our understanding of the data to situations involving more than one fiber characteristic at a time, CCA was used as it simultaneously models effects of multiple independent variables on multiple dependent variables. As CCA uses information from all the variables in both the exposure and outcome variable sets and maximizes the estimation of the relationship between the two sets, CCA may offer a more efficient approach for assessing the relationship of the cell wall probes with fiber characteristics than methods routinely used such as multiple linear regression. CCA starts with simultaneous consideration of both glycan array measurements and the phenotype measures, limiting the inefficiencies that may accompany conventional multiple testing, and thus, reducing type-1 error. The resulting procedure gives a global view of association between indicators of both datasets. Thus, CCA could be used as a comprehensive approach to extract information from data simultaneously. Another major advantage of using the CCA to multiple regression analysis is to deal with the issue of multicollinearity. In multiple regression, the interpretation is usually based on the significance of weights, which is highly influenced by multicollinearity. If two variables have a high correlation one of them will be completely eliminated even if both have a high correlation to the outcome. In our analysis this is illustrated by JIM5 and LM19 (both detecting homogalacturonan), with both showing a high correlation with micronaire in CCA but only LM19 being identified as a predictor of micronaire in the linear regression model. From the results of the CCA, we obtained an overall picture of associations between the glycan and phenotype measurements, with information about the relative contribution of the variables to that particular canonical variate through canonical loadings. The canonical analysis revealed that the canonical correlation was statistically significant at 1%. However, canonical correlation based methods are statistically difficult to assess as they do not fit into a regression framework. In this context penalized CCA adapted with elastic net (CCA-EN) could be used but the elastic net is similar to a lasso soft-thresholding penalization and the algorithm uses partial least squares and not canonical correlation computations [63]. From [63] it is evident that sPLS made a good compromise between all of these approaches and includes variable selection. Additionally, we used the sPLS approach to be able to predict specific cell wall polysaccharides linked with fiber characteristics. Moreover, sPLS maximizes the covariance between the latent variables whereas the canonical correlation based methods maximize the correlation.

There were both unique and common findings from the three types of regression analysis. The major and most significant finding in common to all these analyses is that micronaire is negatively correlated with the xyloglucan (XG) and homogalacturonan (HG) probes. One possible explanation for this observation is that cotton fiber with a high micronaire usually has a very thick secondary cell wall resulting in very high levels of cellulose and lower levels of the non-cellulosic components. However, we do not find a negative correlation of micronaire with other non-cellulosic compounds suggesting that increased cellulose levels of high micronaire fibers affect the XG and HG epitopes in a different way than the other non-cellulosic epitopes. For instance, it could specifically decrease extractability of the XG and HG epitopes. As micronaire measures a combination of fiber fineness and maturity, we wanted to understand whether the observed correlation is with maturity or fineness or a combination of both. We tested this using linear regression models once again and built models for fineness and maturity of the fibers. We observed that the regression models for fineness had an adjusted R^2^ value of 0.803 with JIM5, LM19, and LM25 as significant predictors at a 1% threshold. The regression model for maturity was also significant at the 1% threshold but with no particular significant predictors thereby suggesting that the observed correlation is attributed to fiber fineness. This indicates that this correlation is linked to the thickness rather than the shape of the fiber, which is consistent with a link to the cellulose levels.

Since only the first canonical function of the CCA analysis is statistically significant and this function explains only for micronaire a large fraction of the variance, the results of the CCA analysis are not informative with respect to the other fiber properties. For these fiber properties, the correlation between fiber length and callose is the only one that was detected in both the linear regression and the sPLS analysis. Callose has been described to play a role in cotton fiber elongation. Indeed, it was reported that plasmodesmatal closure was positively correlated with the rapid fiber elongation and that callose was involved in the gating of these plasmodesmata [76]. However, this observation involves transient callose detection, only after 5 dpa and already significantly reduced at 20 dpa, what makes it unlikely to be detected in mature fibers. Other callose deposition was reported by [77]. This callose is supposed to be deposited in the secondary cell wall and remains in the fiber. From the results of the multiple regression models (Table 2), a positive correlation between several of the homogalacturonan probes and length property of the fibers is apparent. The link between pectins and the elongation of cell walls is already observed in several plant systems [78] and studies in flax stems, pea stems and maize coleoptiles revealed a negative correlation between pectin levels and cell elongation. In cotton fibers and trichomes, there exists a positive correlation between pectic sheath and elongation [79] and recent studies by [80] have established that pectic polysaccharides and xyloglucan containing uronic acids were the major polysaccharides extracted during elongation. Hence, our results are in agreement with various studies which state that pectin biosynthesis promotes fiber elongation [81] and that the degree of esterification is a key factor in controlling the elongation [38], [82]. The correlation between length and HG was not detected in the sPLS analysis most likely because the stronger (negative) correlation of HG with micronaire.

Furthermore, relationships between fiber strength or elongation and specific carbohydrate epitopes could be deduced from the results of the sPLS analysis (Figure 2). For instance, fiber strength was associated both with the xylan (LM11) and the extensin (JIM20) epitope. A role of xylan in fiber strength would be consistent with the function of heteroxylan in other cell types which is commonly related to the strengthening of cell walls as revealed by defects in cellulose deposition in xylan mutants [83]. A role of extensin in fiber strength is less expected and would need experimental validation. In the linear regression analysis, extensin was identified as a significant predictor for length AFIS but not for length HVI. A role for extensin in determining cotton fiber length would be more consistent with its role in other plant cell types [84]. Finally, AGP glycan (JIM13) and mannan (BS-400-4 and LM21) epitopes were found to predict cotton fiber elongation from the sPLS model. Interestingly, studies have indicated that AGPs are important players during fiber development. Immunofluorescence assays by JIM 13 showed distinct patterns in developing fiber cells indicating that polysaccharide chains of AGPs are involved in initiation and elongation stages of cotton fibers [85]–[87]. However, it is not clear how these AGPs would affect the elongation property of the mature fiber. These unexpected correlations present thus interesting hypotheses for further structure-function relationship studies of the cotton fiber.

Overall, CoMPP assays of cell wall polysaccharides from cotton fibers suggest that it will be a powerful tool in detecting and quantifying the differences between large sets of cotton lines thus gathering lot of information which is necessary for a proper statistical approach. With the use of predictive statistical approaches to integrate different kinds of datasets, this analysis has thus discovered some correlations that are in line with already known biological functions and others for which the biological relevance still has to be tested. Also, it confirmed the relevance of this type of analysis to enable a detailed understanding of the data from CoMPP assays of cell wall polysaccharides. However, the use of mature cotton fibers in this analysis only allows detecting relevant correlations for components that are still present at maturity. In addition, many changes in polysaccharide composition occur between the fiber elongation stage and maturity. One would thus expect to identify only a fraction of the relationships between polysaccharide composition and fiber properties by analysis of mature fibers, especially for fiber properties such as length that are determined in the early stages of development. Hence it would be interesting to perform a similar kind of analysis using the polysaccharide composition of developing fibers to see whether additional relationships with fiber properties can be determined. The panel of cotton lines used in this study was selected to have maximal diversity in fiber properties and composition. Applying this type of analysis to commercially important cotton lines would allow to understand whether differences in polysaccharide composition affect properties of commercial cotton in the same way as observed in this study and to get insight into the developmental polysaccharides that are essential to obtain high quality cotton fibers. With the sequencing of the *G. hirsutum* genome, cotton fiber research is an exciting field and the work presented here will provide a base for future studies, with potential to translate this study on the developing fibers.

## Supporting Information

Table S1 **Fiber characteristics/phenotype measurements for the 32 cotton lines used in the study.** The plant introduction number (PI number) from the USDA national plant germplasm is also included for each cotton line.
(XLSX)

Table S2 **Comprehensive microarray polymer profiling (CoMPP) heat map of CDTA and NaOH extractions of mature cotton fibers from 32 cotton lines.** References for probe specificity are listed in Table 1.
(XLSX)
